# Long-term comorbidities and mortality associations with treatment in generalized pustular psoriasis: a multi-institutional cohort study

**DOI:** 10.3389/fimmu.2026.1885617

**Published:** 2026-09-03

**Authors:** Teng-Li Lin, Yi-Hsuan Fan, Kuo-Sheng Fan, Chao-Kuei Juan, Yi-Ju Chen, Chun-Ying Wu

**Affiliations:** 1 Department of Dermatology, Dalin Tzu Chi Hospital, Buddhist Tzu Chi Medical Foundation, Chiayi, Taiwan; 2 Ph.D. Program of Interdisciplinary Medicine, National Yang Ming Chiao Tung University, Taipei, Taiwan; 3 Department of Pediatrics, Chung Shan Medical University Hospital, Taichung, Taiwan; 4 Department of Internal Medicine, Division of Chest Medicine, Dalin Tzu Chi Hospital, Buddhist Tzu Chi Medical Foundation, Chiayi, Taiwan; 5 Department of Dermatology, Taichung Veterans General Hospital, Taichung, Taiwan; 6 Department of Post-Baccalaureate Medicine, College of Medicine, National Chung Hsing University, Taichung, Taiwan; 7 Faculty of Medicine and Institute of Clinical Medicine, National Yang Ming Chiao Tung University, Taipei, Taiwan; 8 Institute of Biomedical Informatics, National Yang Ming Chiao Tung University, Taipei, Taiwan; 9 Division of Translational Research, Taipei Veterans General Hospital, Taipei, Taiwan; 10 College of Public Health, China Medical University, Taichung, Taiwan

**Keywords:** biologics, cohort study, comorbidity, generalized pustular psoriasis, mortality, psoriasis, real-world data

## Abstract

**Background:**

Generalized pustular psoriasis (GPP) is a rare, severe inflammatory disease with limited evidence on long-term prognosis and treatment–mortality associations.

**Objective:**

To compare mortality and major comorbidities in GPP, psoriasis vulgaris (PV), and non-psoriatic individuals, and treatment–mortality associations in GPP.

**Methods:**

Adults with GPP, psoriasis vulgaris (PV) without pustular psoriasis, and non-psoriatic controls were identified. GPP patients were 1:1 propensity score–matched to PV and non-psoriatic controls by age, sex, and race. Among GPP patients, those receiving biologics, conventional anti-psoriatic drugs, or small-molecule inhibitors were separately matched to patients treated with systemic corticosteroids alone. Primary outcome was all-cause mortality; secondary outcomes included major mortality-related comorbidities based on the ten leading causes of death per US CDC. Kaplan–Meier and Cox models estimated cumulative incidence and hazard ratios.

**Results:**

After matching, 6,959 GPP–PV and 28,459 GPP–non-psoriatic pairs were analyzed. Over 5 years, GPP patients had higher 5-year mortality than PV (HR, 1.83; 95% CI, 1.28–2.59) and non-psoriatic controls (HR, 2.42; 95% CI, 1.99–2.94). Major comorbidity risks were higher in GPP than in PV, notably ischemic heart disease (HR, 1.34; 95% CI, 1.01–1.78), cerebrovascular disease (HR, 1.62; 95% CI, 1.06–2.47), and neurodegenerative disease (HR, 2.94; 95% CI, 1.47–5.89), and were elevated across all evaluated comorbidities compared with non-psoriatic controls. Mortality risks were particularly pronounced in patients >60 years and males. Among GPP patients, biologic therapy was associated with lower mortality than systemic corticosteroids alone (HR, 0.71; 95% CI, 0.52–0.96), whereas conventional anti-psoriatic drugs and small-molecule inhibitors were not.

**Conclusion:**

GPP is associated with higher mortality and comorbidity risk than PV and non-psoriatic individuals. Biologic therapy was associated with lower mortality.

## 1 Introduction

Generalized pustular psoriasis (GPP) is a rare, severe autoinflammatory skin disease that is increasingly recognized as a distinct clinical entity rather than simply a variant of psoriasis (1, 2). Compared with psoriasis vulgaris (PV), the most prevalent form of psoriasis, GPP shows distinct clinical features and pathophysiology, driven mainly by interleukin (IL)-36 and innate immune dysregulation (3, 4), whereas PV is dominated by IL-23/IL-17–driven adaptive pathways (5). Although approximately half of GPP patients have concomitant PV (1, 2), they are generally considered to have a higher disease burden.

Previous studies have reported higher mortality rates among patients with GPP compared with those with PV, with reported causes of death frequently including sepsis after acute flares and treatment-related complications (1, 2, 6, 7). However, small sample sizes have been a limitation across most studies, resulting in considerable variability in reported mortality rates, ranging from 0% to 16% (1, 2, 7). In addition, long-term major comorbidities beyond those occurring during acute flares in GPP remain poorly understood, and evidence on the prognostic impact of therapies is also limited (8–11). This study aimed to examine mortality, major comorbidities, and therapeutic effects in patients with GPP using a large multi-institutional cohort, compared with PV patients and individuals without psoriasis.

## 2 Methods

### 2.1 Data source

This retrospective cohort study used data from the TriNetX US Collaborative Network, which compiles anonymized, real-world electronic medical records contributed by 71 healthcare organizations, encompassing approximately 131 million patients across the United States (12). The database provides 10–20 years of longitudinal records depending on participating healthcare organizations and is updated approximately every two weeks. Standardized information on demographics, diagnoses, medications, measurements, and procedures is available (**Supplementary eTable 1**) and has been widely used for clinical and epidemiologic research (13, 14). The present analysis was completed on January 12, 2025, using all available longitudinal records within the TriNetX database. The available observation period varied across participating healthcare organizations, with approximately 95% of records occurring between 2007 and 2025. Ethical approval was obtained from Taichung Veterans General Hospital, Taiwan (No. CE23353C), with informed consent waived owing to the use of de-identified data.

### 2.2 Study population

Adults aged ≥18 years were identified from the TriNetX dataset and classified into three groups: GPP (≥3 GPP diagnoses), PV without pustular psoriasis (≥3 PV diagnoses and no GPP, palmoplantar pustular psoriasis, or acrodermatitis continua), and non-PsO (no psoriasis diagnosis). Because GPP diagnosis relies primarily on clinical assessment and lacks universally applied diagnostic criteria, we required at least three separate GPP diagnoses to improve diagnostic specificity and reduce misclassification. The index date was defined as the first relevant diagnosis for GPP or PV patients, and as the first general examination encounter without relevant complaints or diagnoses (International Classification of Diseases, 10th Revision [ICD-10] code Z00) for non-PsO controls. Patients with a diagnosis of other pustulovesicular eruptions (including pompholyx, drug eruption, impetigo, cutaneous candidiasis, or bullous dermatoses) at any time in the database, or with any study outcomes before the index date, were excluded. Diagnoses were identified using standardized ICD-10 codes from routinely collected electronic health records. GPP patients were compared with the PV and non-PsO groups using 1:1 propensity score matching for age, sex, and race.

### 2.3 Covariates

Demographic variables included age, sex, and race. Current age was defined as the patient’s age at the time of data extraction, while age at index referred to their age on the index date. Race information was obtained through patient self-report or clinician assessment and submitted to the TriNetX Network by participating healthcare organizations. These variables were used in the propensity score matching to ensure balanced baseline characteristics across groups, with both current age and age at index included to mitigate potential period effects or calendar-time bias.

### 2.4 Outcomes

The primary outcome was all-cause mortality, identified through healthcare records, insurance claims, and public death registries based on diagnostic codes and reported dates (15). Secondary outcomes included major mortality-related comorbidities. As TriNetX does not provide cause-of-death data, outcomes were selected based on the leading underlying causes of death reported by the U.S. Centers for Disease Control and Prevention (16, 17). Excluding trauma, the ten most common causes in recent years were analyzed: ischemic heart disease, malignant neoplasms, cerebrovascular disease, chronic lower respiratory disease, degenerative nervous system disease, diabetes mellitus, glomerular disease, hepatic fibrosis and cirrhosis, suicidal ideation or suicide attempt, and sepsis. Although additional clinical and metabolic complications following acute GPP flares have been reported in previous studies, the secondary outcomes in this study were pre-specified to evaluate long-term mortality-related disease risks rather than to comprehensively characterize all previously reported GPP-associated comorbidities. Participants were followed for up to five years or until outcome occurrence or last recorded encounter.

### 2.5 Subgroup analyses

Because demographics may be associated with mortality and influence the clinical presentation of GPP (1, 2), we further compared mortality between GPP and PV patients and between GPP and non-PsO patients across subgroups defined by age (18–40, 40–60, and ≥60 years), sex (male and female), and race (White, Black or African American, and Asian).

### 2.6 Sensitivity analyses

Five sensitivity analyses were performed to test the robustness of the mortality comparisons. Model 1 applied a 1-month lag period after the index date, during which deaths were excluded to minimize potential bias from the inclusion of seriously ill patients. Model 2 additionally matched for common pre-existing comorbidities and smoking-related diagnoses—including stroke, hypertension, chronic obstructive pulmonary disease, liver diseases, type 2 diabetes, chronic kidney disease, sleep disorders, obesity, hyperlipidemia tobacco use (ICD-10 Z72.0), and nicotine dependence (ICD-10 F17)—beyond demographic variables to reduce potential confounding. Smoking-related ICD-10 diagnoses were used as proxy indicators of smoking history because direct smoking status is unavailable in the TriNetX database. Model 3 redefined the follow-up starting point for both GPP and PV groups as the first psoriasis diagnosis and matched for both current age and age at index to equalize disease duration between groups. Model 4 excluded patients with any other psoriasis subtypes, retaining only GPP-only and PV-only populations. To preserve adequate sample size, this analysis required only one diagnostic record for disease definition and did not exclude patients with prior diagnoses of the leading underlying causes of death. Model 5 extended the follow-up period from 5 to 10 years while maintaining all other cohort definitions and analytic procedures unchanged”.

### 2.7 Treatment-based analyses

To assess treatment effects on mortality, GPP patients were stratified by systemic therapy initiated within one week of diagnosis. Biologic users included those receiving tumor necrosis factor (TNF)-alpha inhibitors (infliximab, adalimumab, etanercept, golimumab, certolizumab pegol), IL-12/23 inhibitor (ustekinumab), IL-17 inhibitors (secukinumab, ixekizumab, brodalumab), IL-23 inhibitors (guselkumab, tildrakizumab, risankizumab), or IL-36 receptor inhibitor (Spesolimab). Mechanistic class–specific analyses were additionally performed within biologic users. Small-molecule inhibitor users received apremilast or deucravacitinib without biologics, while conventional anti-psoriatic drug users received methotrexate, cyclosporine, or acitretin without biologics or small-molecule agents. These three groups were compared with a reference group who received only systemic corticosteroids but none of the above agents. Covariate matching and follow-up were consistent with the primary mortality analysis, with the index date redefined as treatment initiation.

### 2.8 Statistical analysis

The GPP cohort was compared separately with the PV and non-PsO cohorts using 1:1 propensity score matching without replacement. Propensity scores were estimated by logistic regression, and greedy nearest-neighbor matching with a 0.1 standard deviation (SD) caliper was applied; standardized mean differences <0.1 indicated adequate balance. Kaplan–Meier curves were used to estimate mortality, with group differences assessed by the log-rank test. Hazard ratios (HRs) and 95% confidence intervals (CIs) were estimated using Cox proportional hazards models. Analyses were conducted in R (version 4.3.1), with matching and outcome analyses performed within the TriNetX platform. A two-tailed p <0.05 or 95% CI excluding 1 was considered statistically significant.

## 3 Results

### 3.1 Demographic characteristics

We initially identified 34,372 patients with GPP, 9,920 patients with PV without pustular psoriasis, and 3,068,187 non-psoriatic individuals. After propensity score matching, 6,959 GPP patients were matched to 6,959 PV patients, and 28,459 GPP patients were matched to 28,459 non-PsO controls. Demographic characteristics before and after matching are shown in **Supplementary eTable 2** and **Table 1**, respectively. In both comparisons, the mean age at the index date was approximately 45 years, nearly half of the patients were female, and 60–70% were White. These characteristics were well balanced between groups.

**Table 1:** Demographic characteristics of study subjects after prosperity score matching^a^.

	GPP vs PV			GPP vs non-PsO		
Baseline characteristics	GPP (N = 6,959)	PV (N = 6,959)	Std. diff.	GPP (N = 28,459)	Non-PsO (N = 28,459)	Std. diff.
Age, years, mean ± SD						
Current age	49.95 ± 17.0	50.3 ± 16.3	0.022	53.8 ± 17.0	53.8 ± 17.0	<.001
Age at index	44.6 ± 16.9	44.8 ± 16.2	0.013	43.1 ± 16.9	43.1 ± 16.9	<.001
Female, N (%)	3,612 (51.9%)	3,469 (49.8%)	0.041	13,393 (47.1%)	13,381 (47.0%)	0.001
Race, N (%)^b^						
White	4,857 (69.8%)	5,029 (72.3%)	0.055	18,108 (63.6%)	18,116 (63.7%)	0.001
Black	321 (4.6%)	350 (5.0%)	0.019	1,232 (4.3%)	1,206 (4.2%)	0.005
Asian	350 (5.0%)	358 (5.1%)	0.005	1,021 (3.6%)	1,037 (3.6%)	0.003
Unavailable	1,044(15.0%)	772 (11.1%)	0.116	6993 (24.6%)	7,006 (24.6%)	0.001

GPP, generalized pustular psoriasis; PV, psoriasis vulgaris; PsO, psoriasis; Std. diff., standardized mean difference; SD, standard deviation; N, number.

^a^ Prosperity score matching was conducted based on current age, age at index, sex, and race.

^b^ Races not fully represented include the sparsely numbered American Indian, Alaska Native, Native Hawaiian, and other Pacific Islander.

### 3.2 Mortality and major mortality-related comorbidities

**Figure 1:**
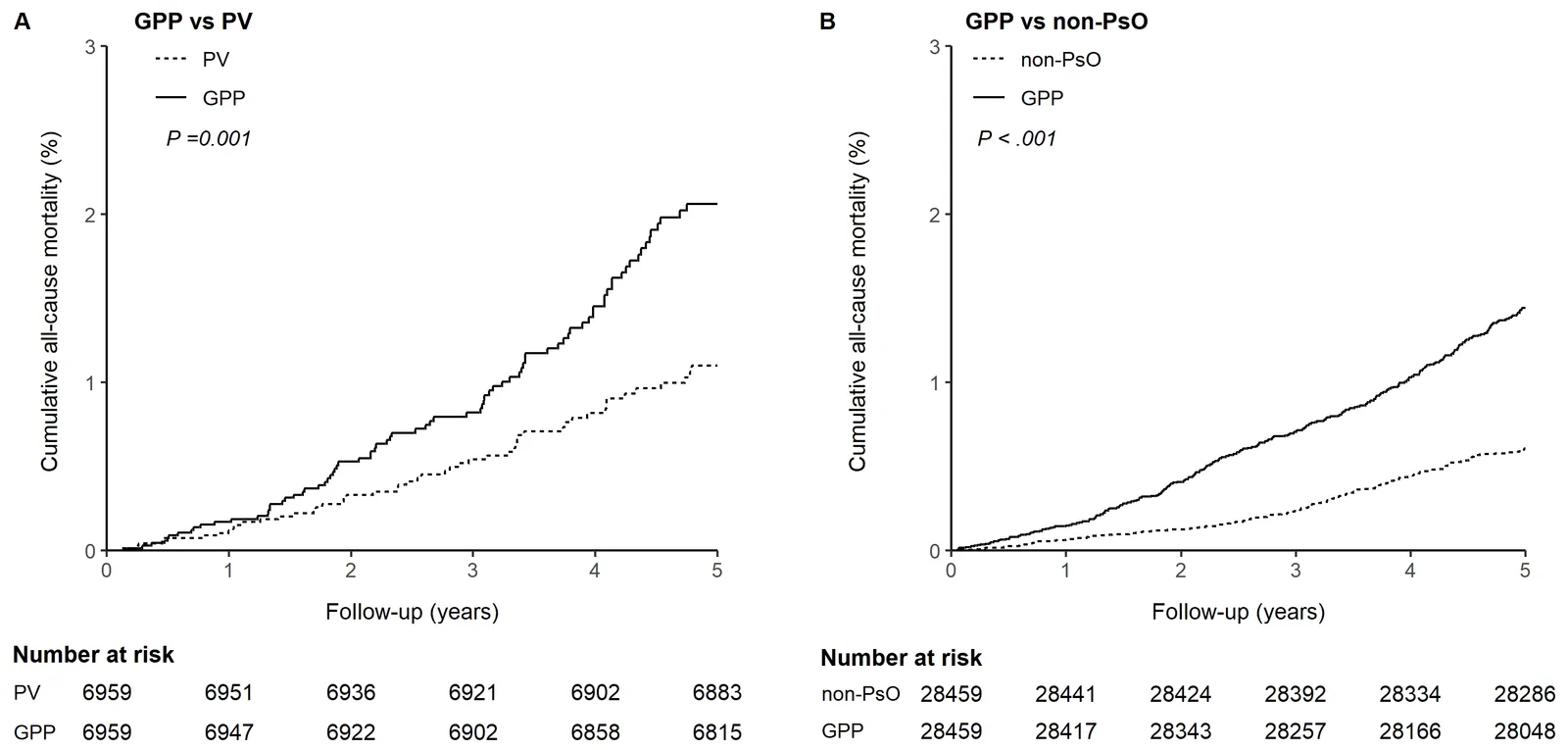
Cumulative incidence of all-cause mortality among **(A)** patients with GPP versus PV and **(B)** patients with GPP versus non-PsO. The differences between the two study cohorts were determined by log-rank test. GPP, generalized pustular psoriasis; PV, psoriasis vulgaris; PsO, psoriasis.

In Kaplan–Meier analysis, the GPP group showed a significantly higher 5-year cumulative all-cause mortality (2.06%; 95% CI, 1.65–2.58) than the PV group (1.10%; 95% CI, 0.83–1.46) (p = .001) (**Figure 1A**). Similarly, GPP patients had a higher 5-year mortality (1.44%; 95% CI, 1.29–1.61) compared with the non-PsO group (0.61%; 95% CI, 0.52–0.71) (p <.001) (**Figure 1B**).

Cox regression revealed increased all-cause mortality in GPP versus PV (HR 1.83; 95% CI, 1.28–2.59), with elevated risks for several major comorbidities, including ischemic heart disease (HR 1.34; 95% CI, 1.01–1.78), cerebrovascular disease (HR 1.62; 95% CI, 1.06–2.47), and degenerative nervous system disease (HR 2.94; 95% CI, 1.47–5.89) (**Table 2**).

**Table 2:** Risk of mortality and major mortality-related comorbidities over 5-year observation for GP patients compared to PV patients and non-PsO patients after prosperity score matching^a^.

	GPP vs PV (N = 6,959 per group)			GPP vs non-PsO (N = 28,459 per group)		
Outcomes	GPP	PV	HR (95% CI)^b^	GPP	Non-PsO	HR (95% CI)^b^
Mortality	82	50	**1.83 (1.28-2.59)**	312	147	**2.42 (1.99-2.94)**
Major mortality-related comorbidities						
Ischemic heart diseases	103	87	**1.34 (1.01-1.78)**	385	129	**3.43 (2.81-4.19)**
Malignant neoplasms	93	85	1.22 (0.91-1.64)	472	197	**2.74 (2.32-3.24)**
Cerebrovascular diseases	52	36	**1.62 (1.06-2.47)**	214	92	**2.65 (2.08-3.39)**
Chronic lower respiratory diseases	154	137	1.26 (1.00-1.58)	721	243	**3.41 (2.95-3.94)**
Degenerative nervous system diseases	29	11	**2.94 (1.47-5.89)**	77	36	**2.44 (1.64-3.63)**
Diabetes mellitus	103	99	1.17 (0.89-1.54)	505	157	**3.68 (3.08-4.40)**
Glomerular diseases	10^c^	0	--	24	10^c^	**3.39 (1.52-7.54)**
Hepatic fibrosis and cirrhosis	19	22	0.96 (0.52-1.78)	91	15	**6.91 (4.00-11.93)**
Suicide	52	47	1.196 (0.81-1.78)	174	150	**1.28 (1.03-1.59)**
Sepsis	51	53	1.04 (0.71-1.53)	232	121	**2.13 (1.71-2.66)**

GPP, generalized pustular psoriasis; PV, psoriasis vulgaris; PsO, psoriasis; HR, hazard ratio; CI, confidence interval.

^a^ Prosperity score matching was conducted based on current age, age at index, sex, and race.

^b^ Bolded HR have 95% CIs that do not include 1.

^c^ To maintain anonymity, TriNetX reports a value of 10 when the number of observations is fewer than 10.

Compared with non-PsO controls, GPP patients had higher all-cause mortality (HR 2.42; 95% CI, 1.99–2.94) and elevated risks across all analyzed major comorbidities, including ischemic heart disease (HR 3.43; 95% CI, 2.81–4.19), malignant neoplasms (HR 2.74; 95% CI, 2.32–3.24), cerebrovascular disease (HR 2.65; 95% CI, 2.08–3.39), chronic lower respiratory disease (HR 3.41; 95% CI, 2.95–3.94), degenerative nervous system disease (HR 2.44; 95% CI, 1.64–3.63), diabetes mellitus (HR 3.68; 95% CI, 3.08–4.40), glomerular disease (HR 3.39; 95% CI, 1.52–7.54), hepatic fibrosis or cirrhosis (HR 6.91; 95% CI, 4.00–11.93), suicide (HR 1.28; 95% CI, 1.03–1.59), and sepsis (HR 2.13; 95% CI, 1.71–2.66) (**Table 2**).

### 3.3 Subgroup analyses

The numbers of deaths and cumulative mortality estimates across subgroups are presented in **Supplementary eTable 3**. GPP patients had higher all-cause mortality than PV across most subgroups, particularly those aged ≥60 years (HR 1.90, 95% CI 1.31–2.76), males (HR 1.87, 95% CI 1.16–3.04), and White patients (HR 1.73, 95% CI 1.17–2.56) (**Table 3**). Compared with non-PsO, GPP patients consistently exhibited elevated mortality risks across all subgroups, with significant increases in patients aged 40–60 years (HR 2.41, 95% CI 1.57–3.72) and ≥60 years (HR 2.07, 95% CI 1.70–2.52), males (HR 2.71, 95% CI 2.07–3.54), females (HR 2.25, 95% CI 1.69–3.00), and White patients (HR 2.33, 95% CI 1.86–2.92) (**Table 3**).

**Table 3:** Subgroup analyses of mortality risk for GPP patients compared to PV patients and non-PsO patients after prosperity score matching^a^.

Subgroups	GPP vs PV	GPP vs Non-PsO
	Hazard ratio (95% CI)^b^	Hazard ratio (95% CI)^b^
By age		
18–40 years	2.59 (0.67-10.02)	1.45 (0.68-3.09)
40–60 years	1.22 (0.50-3.01)	**2.41 (1.57-3.72)**
>60 years	**1.90 (1.31-2.76)**	**2.07 (1.70-2.52)**
Sex		
Male	**1.87 (1.16-3.04)**	**2.71 (2.07-3.54)**
Female	1.24 (0.79-1.95)	**2.25 (1.69-3.00)**
By race		
White	**1.73 (1.17-2.56)**	**2.33 (1.86-2.92)**
Black	0.82 (0.18-3.65)	1.95 (0.96-3.96)
Asian	1.20 (0.17-8.52)	2.85 (0.55-14.69)

GPP, generalized pustular psoriasis; PV, psoriasis vulgaris; PsO, psoriasis; CI, confidence interval.

^a^ Prosperity score matching was conducted based on current age, age at index, sex, and race.

^b^ Bolded hazard ratios have 95% CIs that do not include 1.

### 3.4 Sensitivity analyses

Across all sensitivity analyses, GPP patients consistently exhibited higher all-cause mortality than PV or non-PsO, confirming the robustness of the primary findings (**Supplementary eTable 4**).

### 3.5 Treatment-based analyses

Among GPP patient, biologic therapy was associated with lower all-cause mortality than systemic corticosteroids (HR, 0.71; 95% CI, 0.52–0.96) (**Table 4**). When stratified by mechanism, a significant risk reduction was still observed for IL-12/23 or IL-23 inhibitors (HR, 0.31; 95% CI, 0.10–0.94), but not for TNF-α (HR 1.19; 95% CI 0.80-1.78) or IL-17 (HR 0.41; 95% CI 0.08-2.09) inhibitors. At the time of data extraction, no patients had received IL-36 receptor inhibitors. Conventional anti-psoriatic drugs (HR 1.26; 95% CI, 0.80–1.96) and small-molecule inhibitors (HR 0.30; 95% CI, 0.09–1.07) showed no significant mortality differences versus corticosteroids.

**Table 4:** Comparison of mortality risk between GPP patients receiving non-steroid therapy and systemic corticosteroids after prosperity score matching^a^.

Treatments	Death/N		HR (95% CI)^b^
	Non-steroid therapy	Systemic corticosteroids	
cDMARDs or acitretin^c^	40/1,732	37/1,732	1.26 (0.80-1.96)
Small molecule inhibitors^c^	10/606	12/606	0.30 (0.09-1.07)
Biologics	70/5,945	93/5,945	**0.71 (0.52-0.96)**
TNFi	53/2,638	45/2,638	1.19 (0.80-1.78)
IL17i	10/279^d^	10/279^d^	0.41 (0.08-2.09)
IL12/23i or IL23i	10/912^d^	13/912	**0.31 (0.10-0.94)**

GPP, generalized pustular psoriasis; PV, psoriasis vulgaris; PsO, psoriasis; HR, hazard ratio; CI, confidence interval; cDMARDs, conventional disease-modifying antirheumatic drugs; TNFi, tumor necrosis factor-α inhibitor; IL17i, interleukin-17 inhibitor; IL12/23i, interleukin-12/23 inhibitor; IL23i, interleukin-23 inhibitor.

^a^ Prosperity score matching was conducted based on current age, age at index, sex, and race.

^b^ Bolded hazard ratios have 95% CIs that do not include 1.

^c^ cDMARDs include methotrexate and cyclosporine. Small-molecule inhibitors include apremilast and deucravacitinib.

^d^ To maintain anonymity, TriNetX reports a value of 10 when the number of observations is fewer than 10.

## 4 Discussion

In this multi-institutional retrospective cohort study, GPP patients had higher all-cause mortality than both PV patients without pustular psoriasis and non-PsO individual. Compared with PV patients, risks of ischemic heart disease, cerebrovascular disease, and degenerative nervous system disease were elevated in GPP patients, and risk of all analyzed comorbidities that represent leading causes of death were higher than in non-PsO individuals. Notably, GPP patients receiving biologic therapy had a significantly lower risk of mortality than those treated with systemic corticosteroids.

This study represents one of the largest analyses of GPP to date. The observed 5-year cumulative mortality of ~2% fell within the previously reported range (0–16%) (1, 2, 7), albeit toward the lower end. Broader case capture that included milder GPP phenotypes, the exclusion of patients with baseline comorbidities overlapping with the study outcomes, and the inclusion of more recent data reflecting advances in treatment strategies (18) may collectively explain the relatively lower absolute mortality observed. Despite this, the relative excess risk remained consistent with prior studies (6), with GPP patients showing an 83% higher risk of mortality than PV patients and a 142% higher than individuals without psoriasis. These findings are concordant with our subsequent subgroup mortality analyses and the observed increased risks of multisystem comorbidities in GPP, exceeding those seen not only in non-PsO populations but also in patients with PV.

Among older GPP patients, particularly those aged ≥60 years, both cumulative and relative risk of death were significantly increased. Similar findings were reported by Frysz et al. (7), who observed lower survival among GPP patients older than 55 years, highlighting the adverse prognostic impact of age. Male patients with GPP also exhibited higher mortality risks than their PV and non-psoriatic counterparts, whereas this excess risk was not observed among female patients with GPP versus PV, mirroring prior reports of poorer survival in males and potentially reflecting a protective effect of estrogen on psoriatic inflammation (7, 19–21). Racial differences in the clinical presentation of GPP have been reported (2, 11). While elevated mortality risk was observed among White patients with GPP, results for other racial groups were not definitive, likely due to limited sample sizes.

The clinical, hematological, biochemical, and systemic complications reported in patients with GPP are summarized in **Supplementary Table 5**. Dysregulated IL-36–driven innate immune activation and enhanced NETosis in GPP may promote oxidative stress and endothelial injury, contributing to organ damage and increased risk of death (3, 4, 22). Patients with GPP exhibited an almost sevenfold higher risk of hepatic fibrosis or cirrhosis compared with non-psoriatic individuals, in line with prior reports of frequent liver dysfunction during GPP flares (23). Recurrent IL-36–mediated inflammation with downstream IL-17 amplification may further accelerate atherosclerosis and thromboinflammatory processes (24), potentially contributing to the higher cardiovascular and cerebrovascular risks observed in GPP compared with PV. These findings are consistent with previous research showing a greater burden of hypertension and ischemic heart disease in GPP than in psoriasis (25). Such vascular injury, together with sustained innate immune–mediated neuroinflammation (26), may also underlie the increased risk of neurodegenerative diseases in GPP.

In this study, biologic therapy in patients with GPP was associated with an approximately 30% lower mortality risk compared with systemic corticosteroids, whereas no significant differences were observed for small-molecule inhibitors or conventional anti-psoriatic drugs. This finding aligns with prior research showing lower mortality with biologic therapy than with oral agents or corticosteroids alone in GPP (9). When stratified by mechanism, IL-12/23 or IL-23 inhibitors were associated with a significant mortality reduction, echoing meta-analyses reporting the lowest recurrence rates with IL-23 inhibitors (27). Although IL-36 and IL-17 inhibitors are commonly used in GPP, limited sample sizes in the current cohort precluded robust analyses. Systemic corticosteroids and conventional anti-psoriatic drugs have historically been used in GPP management (28); however, current guidelines generally discourage routine corticosteroid use (29, 30). Moreover, adverse effects of traditional therapies remain important concerns, as prior studies have reported that some GPP-related deaths were attributable to complications associated with these treatments (2).

This study has several strengths. Use of a large real-world, multi-institutional electronic health record database with a stringent case definition enabled identification of a substantial number of patients with GPP, allowing long-term outcome assessment and subgroup analyses in this rare disease. The large sample size also helped reduce selection bias and improve statistical power (12). The robustness of the findings was supported by multiple sensitivity analyses addressing potential sources of bias, including reverse causation (post-index lag period), confounding by major comorbidities and smoking-related diagnoses (additional matching), differences in psoriasis duration (redefinition of follow-up start), and extended follow-up (10-year analysis). Restricting analyses to GPP-only populations further clarified the effects attributable to isolated GPP. In addition, treatment-based analyses adopted a new-user, active-comparator design, minimizing channeling bias and strengthening the validity of comparative mortality estimates. Together, these findings provide additional real-world evidence on the long-term mortality burden, associated disease risks, and treatment-related outcomes of GPP, addressing important gaps in current evidence for this rare disease.

This study has several limitations. First, as an observational study, causal relationships cannot be established. Nevertheless, the results highlight higher mortality and major mortality-related comorbidity risks in GPP compared with PV and non-PsO populations. Second, misclassification is possible, particularly for GPP, given the lack of standardized diagnostic criteria and reliance on clinician judgment and coding (2, 7). To mitigate this, we required at least three GPP diagnoses and excluded other pustulovesicular dermatoses to improve diagnostic specificity. Third, information on lifestyle factors, disease duration and severity, and time-varying medication use was unavailable. In addition, differential healthcare utilization between patients with GPP and comparator groups may have influenced the ascertainment of newly diagnosed outcomes. However, patients with baseline mortality-related comorbidities were excluded, and multiple sensitivity analyses accounting for major comorbidities and disease duration yielded consistent results. Although treatment-based analyses require confirmation with more granular exposure data, our findings provide preliminary evidence suggesting lower mortality associated with biologic therapy. Fourth, cause-specific mortality information was unavailable in the TriNetX database. Therefore, we evaluated incident diseases corresponding to the leading causes of death in the United States as secondary mortality-related disease outcomes to provide insight into long-term mortality-related disease risks associated with GPP. However, because outcomes were selected based on leading causes of death, this study was not intended to provide a comprehensive assessment of all GPP-associated comorbidities. Finally, while a substantial number of U.S. GPP patients were included from the TriNetX database, its open network structure reflects data from participating healthcare organizations (12), which may limit generalizability to other healthcare settings or populations. Nevertheless, the large sample size and multicenter nature of this database enhance statistical power and enable evaluation of outcomes in this rare disease population.

## 5 Conclusions

In conclusion, patients with GPP have higher risks of mortality and major comorbidities than those with PV or without psoriasis. Excess mortality was more pronounced among older patients and males. Among GPP patients, biologic therapy was associated with lower mortality than systemic corticosteroids.

## Data Availability

The original contributions presented in the study are included in the article/**Supplementary Material**. Further inquiries can be directed to the corresponding authors.
