# Correction: Long-term comorbidities and mortality associations with treatment in generalized pustular psoriasis: a multi-institutional cohort study

**DOI:** 10.3389/fimmu.2026.2022137

**Published:** 2026-10-14

**Authors:** Teng-Li Lin, Yi-Hsuan Fan, Kuo-Sheng Fan, Chao-Kuei Juan, Yi-Ju Chen, Chun-Ying Wu

**Affiliations:** 1 Department of Dermatology, Dalin Tzu Chi Hospital, Buddhist Tzu Chi Medical Foundation, Chiayi, Taiwan; 2 Ph.DProgram of Interdisciplinary Medicine, National Yang Ming Chiao Tung University, Taipei, Taiwan; 3 Department of Pediatrics, Chung Shan Medical University Hospital, Taichung, Taiwan; 4 Department of Internal Medicine, Division of Chest Medicine, Dalin Tzu Chi Hospital, Buddhist Tzu Chi Medical Foundation, Chiayi, Taiwan; 5 Department of Dermatology, Taichung Veterans General Hospital, Taichung, Taiwan; 6 Department of Post-Baccalaureate Medicine, College of Medicine, National Chung Hsing University, Taichung, Taiwan; 7 Faculty of Medicine and Institute of Clinical Medicine, National Yang Ming Chiao Tung University, Taipei, Taiwan; 8 Institute of Biomedical Informatics, National Yang Ming Chiao Tung University, Taipei, Taiwan; 9 Division of Translational Research, Taipei Veterans General Hospital, Taipei, Taiwan; 10 College of Public Health, China Medical University, Taichung, Taiwan

**Keywords:** biologics, cohort study, comorbidity, generalized pustular psoriasis, mortality, psoriasis, real-world data

An incorrect **Funding** statement was provided. The correct funder is “Ministry of Science and Technology, Taiwan”. An incorrect number was provided for the funder Ministry of Science and Technology, Taiwan. The correct number is “MOST 114-2314-B-005-001-MY3”.

An incorrect number was provided for funder Taichung Veterans General Hospital. The correct number is “TCVGH 1155602D”. The correct **Funding** statement reads:

“The author(s) declared that financial support was received for this work and/or its publication. This work is supported in part by the Ministry of Science and Technology, Taiwan (MOST 114-2314-B-005-001-MY3) and Taichung Veterans General Hospital (TCVGH 1155602D). The funders of the study had no involvement in the design and conduct of the study; collection, management, analysis, and interpretation of the data; preparation, review, or approval of the manuscript; and decision to submit the manuscript for publication.”

The original version of this article has been updated.

