# Supplementary material for: Long-term comorbidities and mortality associations with treatment in generalized pustular psoriasis: a multi-institutional cohort study
Source: Front Immunol. 2026 Sep 3;17:1885617. doi: 10.3389/fimmu.2026.1885617 (PMC13582526; doi:10.3389/fimmu.2026.1885617)
Supplement: Supplementary file 1 [file Table1.docx]

Supplementary materials for

Long-Term Comorbidities and Mortality Associations with Treatment in Generalized Pustular Psoriasis: A Multi-Institutional Cohort Study

Teng-Li Lin, Yi-Hsuan Fan, Kuo-Sheng Fan, Chao-Kuei Juan, Yi-Ju Chen*, Chun-Ying Wu*

*Corresponding author: [yjchenmd@vghtc.gov.tw](mailto:yjchenmd@vghtc.gov.tw) (Chen YJ) and [cywu4@ym.edu.tw](mailto:cywu4@ym.edu.tw) (Wu CY)

**This file includes:**

- Supplementary Table 1. Codes for all diagnoses and medications in our analysis
- Supplementary Table 2. Demographic characteristics of study subjects before prosperity score matching
- Supplementary Table 3. Subgroup analyses of mortality risk over 5 years for GPP patients compared to PV patients and non-PsO patients after prosperity score matching
- Supplementary Table 4. Sensitivity analyses of mortality risk for GPP patients compared to PV patients and non-PsO patients after prosperity score matching
- Supplementary Table 5. Comorbidities reported in generalized pustular psoriasis and mortality-related disease outcomes evaluated in the present study

| Supplementary Table 1. Codes for all diagnoses and medications in our analysis | | | | |
| --- | --- | --- | --- | --- |
| Specific data | | | | Standardized code |
| Diagnoses | | | | ICD-10-CM code |
|  | Psoriasis | | | L40 |
|  |  | Psoriasis vulgaris | | L40.0 |
|  |  | Pustular psoriasis | | L40.1-L40.3 |
|  |  |  | Generalized pustular psoriasis | L40.1 |
|  |  |  | Acrodermatitis continua | L40.2 |
|  |  |  | Palmoplantar pustular psoriasis | L40.3 |
|  | Encounter for general examination without complaint, suspected or reported diagnosis | | | Z00 |
|  | Pompholyx | | | L30.1 |
|  | Generalized skin eruption due to drugs and medicaments taken internally | | | L27.0 |
|  | Impetigo | | | L01 |
|  | Cutaneous candidiasis | | | B37.2 |
|  | Bullous dermatoses | | | L10-L14 |
|  | Ischemic heart disease | | | I20-I25 |
|  | Malignant neoplasms | | | C00-C26, C30-C58, C60-96, C7A-C7A, or C7B-C7B |
|  | Cerebrovascular disease (stroke) | | | I60-I69 |
|  | Chronic lower respiratory disease | | | J40-J4A |
|  | Degenerative nervous system disease | | | G30-G32 |
|  | Diabetes mellitus | | | E08-E13 |
|  | Glomerular disease | | | N00-N08 |
|  | Hepatic fibrosis and cirrhosis | | | K74 |
|  | Suicidal ideation or suicide attempt | | | R45.851, T14.91, T14.91XA, T14.91XD, or T14.91XS |
|  | Sepsis | | | A40, A41, R65.2 |
|  | Hypertension | | | I10 |
|  | Chronic obstructive pulmonary disease | | | J44 |
|  | Liver diseases | | | K70−77 |
|  | Type 2 diabetes | | | E11 |
|  | Chronic kidney disease | | | N18 |
|  | Sleep disorders | | | G47 |
|  | Obesity | | | E66 |
|  | Hyperlipidemia | | | E78.5 |
|  | Tobacco use | | | Z72.0 |
|  | Nicotine dependence | | | F17 |
| Medications | | | | RxNorm or ATC code |
|  | Systemic corticosteroids | | | ATC:H02 |
|  | Conventional anti-psoriatic drugs | | |  |
|  |  | Methotrexate | | RxNorm:6851 |
|  |  | Cyclosporine | | RxNorm:3008 |
|  |  | Acitretin | | RxNorm:16818 |
|  | Small-molecule inhibitors | | |  |
|  |  | Apremilast | | RxNorm:1492727 |
|  |  | Deucravacitinib | | RxNorm:2612087 |
|  | Biologics | | |  |
|  |  | Tumor necrosis factor-alpha inhibitors | |  |
|  |  |  | Infliximab | RxNorm:191831 |
|  |  |  | Adalimumab | RxNorm:327361 |
|  |  |  | Etanercept | RxNorm:214555 |
|  |  |  | Golimumab | RxNorm:819300 |
|  |  |  | Certolizumab pegol | RxNorm:OMOP5175190 |
|  |  | Interleukin-12/23 inhibitor | |  |
|  |  |  | Ustekinumab | RxNorm:847083 |
|  |  | Interleukin-17 inhibitors | |  |
|  |  |  | Secukinumab | RxNorm:1599788 |
|  |  |  | Ixekizumab | RxNorm:1745099 |
|  |  |  | Brodalumab | RxNorm:1872251 |
|  |  | Interleukin-23 inhibitors | |  |
|  |  |  | Guselkumab | RxNorm:1928588 |
|  |  |  | Tildrakizumab | RxNorm:2053436 |
|  |  |  | Risankizumab | RxNorm:2166040 |
|  |  | Interleukin-36 inhibitors | |  |
|  |  |  | Spesolimab | RxNorm:2610417 |
| ICD-10-CM, International Classification of Diseases, Tenth Revision, Clinical Modification; RxNorm, A standardized drug naming system from the U.S. National Library of Medicine; ATC, Anatomical Therapeutic Chemical Classification System. | | | | |

| Supplementary Table 2. Demographic characteristics of study subjects before prosperity score matching | | | | | | | |
| --- | --- | --- | --- | --- | --- | --- | --- |
|  | | GPP (N = 34,372) | PV (N = 9,920) | | | Non-PsO (N = 3,068,187) | |
| Age, years, mean±SD | |  | |  |  | |  |
|  | Current age | 53.8±17.0 | 49.8±16.3 | | | 46.2±18.5 | |
|  | Age at index | 43.1±16.9 | 44.9±16.2 | | | 37.6±19.3 | |
| Female, N (%) | | 13,393 (47.1%) | 4,799 (48.4%) | | | 1,635,071 (56.0%) | |
| Race, N (%)^a^ | |  |  | | |  | |
|  | White | 18,108 (63.6%) | 7,352 (74.2%) | | | 1,899,274 (65.0%) | |
|  | Black | 1,232 (4.3%) | 494 (5.0%) | | | 355,279 (12.2%) | |
|  | Asian | 1,021 (3.6%) | 515 (5.2%) | | | 159,531 (5.5%) | |
|  | Unavailable | 6,994 (24.6%) | 902 (9.1%) | | | 358,353 (12.3%) | |
| GPP, generalized pustular psoriasis; PV, psoriasis vulgaris; PsO, psoriasise; SD, standard deviation; N, number.  ^a^Races not fully represented include the sparsely numbered American Indian, Alaska Native, Native Hawaiian, and other Pacific Islander. | | | | | | | |

| Supplementary Table 3. Subgroup analyses of mortality risk over 5 years for GPP patients compared to PV patients and non-PsO patients after prosperity score matching^a^ | | | | | | | | |
| --- | --- | --- | --- | --- | --- | --- | --- | --- |
|  | | GPP vs PV | | |  | GPP vs non-PsO | | |
|  | | Death/N | Cumulative mortality | p-value |  | Death/N | Cumulative mortality | p-value |
| By age | |  |  | |  |  |  |  |
|  | 18-40 years | 10/2,308 vs 10/2,308^b^ | 0.60% vs 0.29% | 0.152 |  | 15/7,303 vs 12/7,303 | 0.29% vs 0.20% | 0.339 |
|  | 40-60 years | 10/2,843 vs 10/2,308^b^ | 0.65% vs 0.43% | 0.661 |  | 65/12,537 vs 30/12,537 | 0.66% vs 0.27% | <.001 |
|  | >60 years | 76/2,283 vs 44/2,283 | 5.21% vs 2.87% | 0.001 |  | 282/12,452 vs 150/12,452 | 2.76% vs 1.37% | <.001 |
| Sex | |  |  |  |  |  |  |  |
|  | Male | 45/3,089 vs 26/3,089 | 2.40% vs 1.38% | 0.010 |  | 183/13,383 vs 76/13,383 | 1.75% vs 0.65% | <0.01 |
|  | Female | 40/3,683 vs 35/3,683 | 2.03% vs 1.47% | 0.349 |  | 140/14,809 vs 70/14,809 | 1.22% vs 0.54% | <.001 |
| By race | |  |  |  |  |  |  |  |
|  | White | 67/4,944 vs 40/4,944 | 2.20% vs 1.29% | 0.006 |  | 238/20,108 vs 113/20,108 | 1.46% vs 0.64% | <.001 |
|  | Black or African American | 10/304 vs 10/304^b^ | 1.60% vs 1.90% | 0.789 |  | 21/1,233 vs 12/1,233 | 2.14% vs 1.12% | 0.061 |
|  | Asian | 10/379 vs 10/379^b^ | 1.10% vs 0.62% | 0.856 |  | 10/1,139 vs 10/1,139^b^ | 0.57% vs 0.22% | 0.191 |
| GPP, generalized pustular psoriasis; PV, psoriasis vulgaris; PsO, psoriasis; N., number per subrogup; HR, hazard ratio; CI, confidence interval.  ^a^Prosperity score matching was conducted based on current age, age at index, sex, and race.  ^b^To maintain anonymity, TriNetX reports a value of 10 when the number of observations is fewer than 10. | | | | | | | | |

| Supplementary Table 4. Sensitivity analyses of mortality risk for GPP patients compared to PV patients and non-PsO patients after prosperity score matching^a^ | | | |
| --- | --- | --- | --- |
| Sensitivity model^b^ | GPP vs PV |  | GPP vs Non-PsO |
|  | Hazard ratio (95% CI) |  | Hazard ratio (95% CI) |
| Model 1 | 1.83 (1.28-2.59) |  | 2.40 (1.97-2.92) |
| Model 2 | 1.49 (1.06-2.10) |  | 1.34 (1.12-1.59) |
| Model 3 | 2.18 (1.48-3.21) |  | 2.65 (2.13-3.29) |
| Model 4 | 2.21 (1.34-3.66) |  | 2.55 (1.59-4.09) |
| Model 5 | 1.42 (1.08, 1.86) |  | 1.51 (1.34, 1.70) |
| GPP, generalized pustular psoriasis; PV, psoriasis vulgaris; PsO, psoriasis; CI, confidence interval.  ^a^Prosperity score matching was conducted based on current age, age at index, sex, and race.  ^b^Four sensitivity analyses were performed: Model 1 excluded deaths within 1 month after the index date; Model 2 additionally matched major pre-existing comorbidities and smoking history; Model 3 redefined follow-up from the first psoriasis diagnosis with age matching; Model 4 restricted analyses to GPP-only and PV-only populations; and Model 5 extended follow-up to 10 years for all-cause mortality. | | | |

| Supplementary Table 5. Clinical, hematological, biochemical, and systemic complications reported in patients with generalized pustular psoriasis | | | |
| --- | --- | --- | --- |
| Category | | Complications/abnormalities | References |
| Clinical | | Fever, general malaise, fatigue, disabling edema, arthralgia, generalized pustules, mucosal involvement (cheilitis, geographic or fissured tongues, and genital lesions) | S1-S4 |
| Hematological/ biochemical | | Leukocytosis, neutrophilia, hypocalcemia, hypoalbuminemia, elevated CRP levels, abnormal liver function tests | S1-S4 |
| Systemic/organ | |  |  |
|  | Cardiovascular | Hypertension, ischemic heart disease^a^, congestive heart failure, cerebrovascular disease^a^ | S5-S13, present study |
|  | Respiratory | Chronic obstructive pulmonary disease, interstitial pneumonia, chronic lower respiratory disease^a^ | S8, S9, S12, S14, present study |
|  | Metabolic | Diabetes mellitus^a^, hyperlipidaemia | S5-S13, S15, present study |
|  | Renal | Renal dysfunction, glomerular disease^a^ | S6, S9, S12, S16, present study |
|  | Gastrointestinal | Neutrophilic cholangitis, hepatic fibrosis/cirrhosis^a^ | S17-19, present study |
|  | Infection | Sepsis^a^ | S7, S12, S20, present study |
|  | Neurological | Neurodegenerative disease^a^ | Present study |
|  | Musculoskeletal | Arthritis, psoriatic arthritis | S5, S7, S9, S10, S12, S13, S17, S20-S29 |
|  | Psychiatric | Anxiety, depression, suicide^a^ | S9, S12, present study |
|  | Malignancy | Malignant neoplasms^a^ | Present study |
| References cited in this table are listed separately in the Supplementary References and are numbered independently from those in the main manuscript.  ^a^Complications corresponding to mortality-related disease outcomes evaluated in the present study. | | | |

**Supplementary References**

S1.	Takeichi T, Akiyama M. Generalized Pustular Psoriasis: Clinical Management and Update on Autoinflammatory Aspects. *Am J Clin Dermatol* 2020;21(2):227-36.

S2.	Choon SE, Navarini AA, Pinter A. Clinical Course and Characteristics of Generalized Pustular Psoriasis. *Am J Clin Dermatol* 2022;23(Suppl 1):21-9.

S3.	Hoegler KM, John AM, Handler MZ, Schwartz RA. Generalized pustular psoriasis: a review and update on treatment. *J Eur Acad Dermatol Venereol* 2018;32(10):1645–51.

S4.	Prinz JC, Choon SE, Griffiths CEM, Merola JF, Morita A, Ashcroft DM, Viguier M. Prevalence, comorbidities and mortality of generalized pustular psoriasis: A literature review. *J Eur Acad Dermatol Venereol* 2023;37(2):256-73.

S5.	Imafuku S, Honma M, Okubo Y, Komine M, Ohtsuki M, Morita A, et al. Efficacy and safety of secukinumab in patients with generalized pustular psoriasis: A 52-week analysis from phase III open-label multicenter Japanese study. *J Dermatol* 2016;43(9):1011-7.

S6.	Morita A, Yamazaki F, Matsuyama T, Takahashi K, Arai S, Asahina A, et al. Adalimumab treatment in Japanese patients with generalized pustular psoriasis: Results of an open-label phase 3 study. *J Dermatol* 2018;45(12):1371-80.

S7.	Miyachi H, Konishi T, Kumazawa R, Matsui H, Shimizu S, Fushimi K, et al. Treatments and outcomes of generalized pustular psoriasis: A cohort of 1516 patients in a nationwide inpatient database in Japan. *J Am Acad Dermatol* 2022;86(6):1266-74.

S8.	Okubo Y, Kotowsky N, Gao R, Saito K, Morita A. Clinical characteristics and health-care resource utilization in patients with generalized pustular psoriasis using real-world evidence from the Japanese Medical Data Center database. *J Dermatol* 2021;48(11):1675-87.

S9.	Kromer C, Loewe E, Schaarschmidt ML, Pinter A, Gerdes S, Herr R, et al. Poortinga S, Moessner R, Wilsmann-Theis D. Drug survival in the treatment of generalized pustular psoriasis: A retrospective multicenter study. *Dermatol Ther* 2021;34(2):e14814.

S10.	Choon SE, Lai NM, Mohammad NA, Nanu NM, Tey KE, Chew SF. Clinical profile, morbidity, and outcome of adult-onset generalized pustular psoriasis: analysis of 102 cases seen in a tertiary hospital in Johor, Malaysia. *Int J Dermatol* 2014;53(6):676-84.

S11.	Twelves S, Mostafa A, Dand N, Burri E, Farkas K, Wilson R, et al. Clinical and genetic differences between pustular psoriasis subtypes. *J Allergy Clin Immunol* 2019;143(3):1021-26.

S12.	Hanna ML, Singer D, Bender SD, Valdecantos WC, Wu JJ. Characteristics of hospitalizations and emergency department visits due to generalized pustular psoriasis in the United States. *Curr Med Res Opin* 2021;37(10):1697-703.

S13.	Viguier M, Bentayeb M, Azzi J, de Pouvourville G, Gloede T, Langellier B, et al. Generalized pustular psoriasis: A nationwide population-based study using the National Health Data System in France. *J Eur Acad Dermatol Venereol* 2024;38(6):1131-39.

S14.	Morita A, Kotowsky N, Gao R, Shimizu R, Okubo Y. Patient characteristics and burden of disease in Japanese patients with generalized pustular psoriasis: Results from the Medical Data Vision claims database. *J Dermatol* 2021;48(10):1463-73.

S15.	Youshou G, Wenyan X, Zhiqiang C. Evaluation of the effectiveness of various pustular psoriasis treatments in 90 cases. *Journal of Dermatological Treatment,* 1994;5(1):33–35.

S16.	Hu Y, Yin L, Xu J, Yin Z. Renal function of psoriatic patients: erythrodermic psoriasis has more significant hyperuricemia. *Biomed Res* 2017;28:2515– 8.

S17.	Viguier M, Allez M, Zagdanski AM, Bertheau P, de Kerviler E, Rybojad M, et al. High frequency of cholestasis in generalized pustular psoriasis: Evidence for neutrophilic involvement of the biliary tract. *Hepatology* 2004;40(2):452-8.

S18.	Chandran NS, Chong WS. A dramatic response to a single dose of infliximab as rescue therapy in acute generalized pustular psoriasis of von Zumbusch associated with a neutrophilic cholangitis. *Australas J Dermatol* 2010;51(1):29-31.

S19.	Allez M, Roux ME, Bertheau P, Erlinger S, Degott C, Morel P, et al. Recurrent cholestatic jaundice associated with generalized pustular psoriasis: evidence for a neutrophilic cholangitis. *J Hepatol* 2000;33(1):160-2.

S20.	Wang Q, Liu W, Zhang L. Clinical features of von Zumbusch type of generalized pustular psoriasis in children: a retrospective study of 26 patients in southwestern China. *An Bras Dermatol* 2017;92(3):319-22.

S21.	Hayashi M, Nakayama T, Hirota T, Saeki H, Nobeyama Y, Ito T, et al. Novel IL36RN gene mutation revealed by analysis of 8 Japanese patients with generalized pustular psoriasis. *J Dermatol Sci* 2014;76(3):267-9.

S22.	Yamasaki K, Nakagawa H, Kubo Y, Ootaki K; Japanese Brodalumab Study Group. Efficacy and safety of brodalumab in patients with generalized pustular psoriasis and psoriatic erythroderma: results from a 52-week, open-label study. *Br J Dermatol* 2017;176(3):741-51.

S23.	Sano S, Kubo H, Morishima H, Goto R, Zheng R, Nakagawa H. Guselkumab, a human interleukin-23 monoclonal antibody in Japanese patients with generalized pustular psoriasis and erythrodermic psoriasis: Efficacy and safety analyses of a 52-week, phase 3, multicenter, open-label study. *J Dermatol* 2018;45(5):529-39.

S24.	Borges-Costa J, Silva R, Gonçalves L, Filipe P, Soares de Almeida L, Marques Gomes M. Clinical and laboratory features in acute generalized pustular psoriasis: a retrospective study of 34 patients. *Am J Clin Dermatol* 2011;12(4):271-6.

S25.	Jin H, Cho HH, Kim WJ, Mun JH, Song M, Kim HS, et al. Clinical features and course of generalized pustular psoriasis in Korea. *J Dermatol* 2015;42(7):674-8.

S26.	Wang Y, Cheng R, Lu Z, Guo Y, Yan M, Liang J, et al. Clinical profiles of pediatric patients with GPP alone and with different IL36RN genotypes. *J Dermatol Sci* 2017;85(3):235-40.

S27.	Lau BW, Lim DZ, Capon F, Barker JN, Choon SE. Juvenile generalized pustular psoriasis is a chronic recalcitrant disease: an analysis of 27 patients seen in a tertiary hospital in Johor, Malaysia. *Int J Dermatol* 2017;56(4):392-99.

S28.	Mössner R, Wilsmann-Theis D, Oji V, Gkogkolou P, Löhr S, Schulz P, et al. The genetic basis for most patients with pustular skin disease remains elusive. *Br J Dermatol* 2018;178(3):740-48.

S29.	Tay YK, Tham SN. The profile and outcome of pustular psoriasis in Singapore: a report of 28 cases. *Int J Dermatol* 1997;36(4):266-71.
